# The Equilibria of Diosgenin–Phosphatidylcholine and Diosgenin–Cholesterol in Monolayers at the Air/Water Interface

**DOI:** 10.1007/s00232-016-9914-1

**Published:** 2016-06-27

**Authors:** Katarzyna Janicka, Izabella Jastrzebska, Aneta Dorota Petelska

**Affiliations:** Institute of Chemistry, University of Bialystok, K. Ciolkowskiego 1K, 15-245 Bialystok, Poland

**Keywords:** Diosgenin, Phosphatidylcholine, Cholesterol, Complex formation equilibria, Monolayer, Langmuir trough

## Abstract

Diosgenin (Dio) has shown many treatment properties, but the most important property is cytotoxic activity in cancer cells. In this study, we investigated monolayers of Dio, cholesterol (Ch), and phosphatidylcholine (PC) at the air/water interface. The measurements were carried with a Langmuir Teflon trough and a Nima 9000 tensiometer program. The surface tension values of pure and mixed monolayers were used to calculate π–A isotherms and determine molecular surface areas. We were able to demonstrate the formation of complexes between Dio and PC and Dio and Ch molecules also. We considered the equilibrium between individual components and the formed complexes. In addition, we established that diosgenin and the lipids formed highly stable 1:1 complexes.

## Introduction

Since the dawn of time, the plants were the main source of drugs. The World Health Organization (WHO) announced the list of 21,000 plants, which have medicinal effects worldwide (Patel et al. 2012). One of the medicinal plants species are saponins which can be found in Agavaceae, Dioscoreaceae, Liliaceae, Solanaceae, Scrophulariaceae, Amaryryllidaceae, Leguminosae, and Rhamnaceae (Chen et al. 2015). The Dioscoreaceae Family is known as yams. Plants of genus *Dioscorea* are an essential component of food for many populations in Africa, Asia, and tropical America. The leading production comes from West Africa contributing to 95 % of the world production (Contreras–Pacheco et al. 2013).

**Fig. 1:**
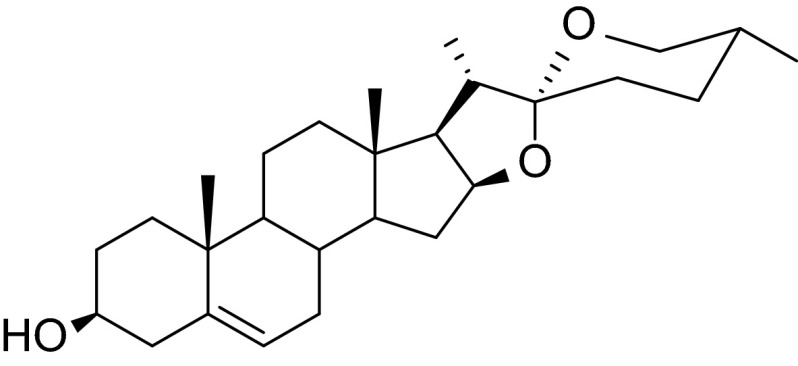
Diosgenin structure

For the first time, diosgenin (Dio) was discovered in 1935 by Fujii and Matsukawa in *Dioscorea Tokoro Makino*. This substance is a steroidal sapogenin predominantly found in *yams*, fenugreek, and *Costus speciosus*. Dio molecule (Fig. 1) is structurally similar to cholesterol (Ch) and other steroids (Hostettmann and Marston 1995). In the pharmaceutical industry, Dio is a very important source of steroidal hormones. The saponin is a precursor of sex hormones (progesterone), corticosteroids (corticosone), and contraceptives (Djerassi et al. 1952). Many studies have shown that Dio plays an important role in Ch metabolism (Liu et al. 2005, Chen et al. 2015). Dio has both antioxidant property and anticholesterolomic activity (Selim and Jaouni 2015). Dio has been used as an antihypercholesterolemia, antihypertriacylglycerolemia, antidiabetes, and antihyperglicemia agent (Chen et al. 2011). In addition, Dio possesses anticancer properties. Many studies showed that Dio inhibits proliferation and induces apoptosis in a variety of tumor cells such as osteosarcoma, colon carcinoma, hepatoma, leukemia, and breast carcinoma (Moalic et al. 2001; Chiang et al. 2007; Li et al. 2010; He et al. 2014).

The objective of the present work was to examine the possible effect of Dio on the lipid monolayer from phosphatidylcholine (PC) or Ch. The molecular interactions between Dio and PC and Dio and Ch were also studied. The interaction was checked by analyzing physicochemical properties for binary mixed monolayers (lipid–Dio), treated as the simplest model of a half of the biological membrane. In this paper, we present evidence for the formation of 1:1 PC–Dio and Ch–Dio complexes at the air/water interface and calculate their stability constants and Gibbs free energy values.

Understanding the physical chemistry of interactions between naturally occurring substances (e.g., Dio) and cell membranes is an important topic, in view of the very high number of possible applications in fields such as drug discovery. Studies on experimental models are an invaluable source of knowledge about the processes occurring in natural membranes. Therefore, the results of our studies have potential implications for both pharmaceutical research and medical diagnostics.

## Theory

The individual components: denoted by L (phosphatidylcholine (PC) or cholestrol (Ch)) and Dio (diosgenin) can form complexes. Two substances can form complexes of varying stoichiometry. However, due to the fact that the first stability constant in complexes is usually the largest (Inczedy 1976), we assumed that 1:1 complexes were predominant.

The equilibrium of complexation reaction (1:1 complex) presented below:

$${\text{L}} + {\text{Dio}} \rightleftarrows {\text{L}} - {\text{Dio}}$$

and may be described by the system of equations (Petelska and Figaszewski 2009):

1 $$a_{\text{L}} S_{\text{L}} + a_{\text{Dio}} S_{\text{Dio}} + a_{{{\text{L}} - {\text{Dio}}}} S_{{{\text{L}} - {\text{Dio}}}} = 1,$$

2 $$a_{\text{L}} + a_{{{\text{L}} - {\text{Dio}}}} = c_{\text{L}} ,$$

3 $$a_{\text{Dio}} + a_{{{\text{L}} - {\text{Dio}}}} = c_{\text{Dio}} ,$$

4 $$K_{{{\text{L}} - {\text{Dio}}}} = \frac{{a_{{{\text{L}} - {\text{Dio}}}} }}{{a_{\text{L}} \cdot a_{\text{Dio}} }},$$

5 $$x_{\text{Dio}} = \frac{{c_{\text{Dio}} }}{{c_{\text{L}} + c_{\text{Dio}} }},$$

where *a*_L_, *a*_Dio_, and *a*_L–Dio_ (mol m^−2^) are the surface concentrations of components L and Dio and complex L–Dio; *c*_L_ and *c*_Dio_ (mol m^−2^) are the total surface concentrations of components L and Dio; *S*_L_, *S*_Dio_, and *S*_L–Dio_ (m^2^ mol^−1^) are the surface areas occupied by 1 mol of components L and Dio and complex L–Dio; *K*_L–Dio_ (m^2^ mol^−1^) is the stability constant of complex L–Dio, and *x*_L_ and *x*_Dio_ are the mole fractions of components L and Dio.

The system of Eqs. (1)–(5) contains unknown quantities *a*_L_, *a*_Dio_, *a*_L–Dio_, *S*_L–Dio_, and *K*_L–Dio_ as well as known or easy to determined quantities *S*_L_, *S*_Dio_, *x*_Dio_, *c*_L_, and *c*_Dio_.

Attempts to solve this system of equations resulted in complicated expressions, so Eqs. (1)–(5) were differentiated with respect to *x*_Dio_ and approximated to low or high argument values. At *x*_Dio_ → 0 (a monolayer formed from pure component L; *x*_L_ → 1), the system of equations is simplified to

6 $$a_{\text{L}}^{'} S_{\text{L}} + a_{\text{Dio}}^{'} S_{\text{Dio}} + a_{{{\text{L}} - {\text{Dio}}}}^{'} S_{{{\text{L}} - {\text{Dio}}}} = 0,$$

7 $$a_{\text{L}}^{ '} + a_{{{\text{L}} - {\text{Dio}}}}^{'} = c_{{{\text{L}}(x_{\text{Dio}} = 0)}}^{'} ,$$

8 $$a_{\text{Dio}}^{ '} + a_{{{\text{L}} - {\text{Dio}}}}^{'} = c_{{{\text{Dio}}(x_{\text{Dio}} = 0)}}^{'} ,$$

9 $$a_{{{\text{L}} - {\text{Dio}}}}^{'} = K_{{{\text{L}} - {\text{Dio}}}} \frac{1}{{S_{\text{L}} }}a_{\text{Dio}}^{'} .$$

At *x*_Dio_ → 1 (a monolayer formed from pure component Dio; *x*_L_ → 0), the system of equations after differentiation with respect to *x*_Dio_ is simplified to

10 $$a_{\text{L}}^{ '} S_{\text{L}} + a_{\text{Dio}}^{ '} S_{\text{Dio}} + a_{{{\text{L}} - {\text{Dio}}}}^{'} S_{{{\text{L}} - {\text{Dio}}}} = 0,$$

11 $$a_{\text{L}}^{ '} + a_{{{\text{L}} - {\text{Dio}}}}^{'} = c_{{{\text{L}}(x_{\text{Dio}} = 1)}}^{'} ,$$

12 $$a_{\text{Dio}}^{ '} + a_{{{\text{L}} - {\text{Dio}}}}^{'} = c_{{{\text{Dio}}(x_{\text{Dio}} = 1)}}^{'} ,$$

13 $$a_{{{\text{L}} - {\text{Dio}}}}^{'} = K_{{{\text{L}} - {\text{Dio}}}} \frac{1}{{S_{\text{Dio}} }}a_{\text{L}}^{ '} .$$

In the above equations, $a_{\text{L}}^{\prime }$, $a_{Dio}^{\prime }$ and $a_{{{\text{L}} - {\text{Dio}}}}^{\prime }$ are the derivatives of *a*_L_, *a*_Dio_, and *a*_L–Dio_ with respect to *x*_Dio_.

The quantities $a_{\text{L}}^{\prime }$, $a_{Dio}^{\prime }$ and $a_{{{\text{L}} - {\text{Dio}}}}^{\prime }$ may be eliminated from the system of equations if the values of S_L_ and S_Dio_ are known. Suitable transformations lead to expressions for two quantities of interest (each individually): the stability constant of the complex *K*_L–Dio_ and the surface area occupied by one molecule of the complex *S*_L–Dio_:

14 $$K_{{{\text{L}} - {\text{Dio}}}} = \frac{{S_{\text{Dio}}^{3} c_{{{\text{Dio}}(x_{\text{Dio}} = 1)}}^{'} - 2S_{\text{L}} S_{\text{Dio}} - S_{\text{L}}^{3} c_{{{\text{L}}(x_{\text{Dio}} = 0)}}^{'} }}{{S_{\text{Dio}} - S_{\text{L}} + S_{\text{L}}^{ 2} c_{{{\text{L}}(x_{\text{Dio}} = 0)}}^{'} + S_{\text{Dio}}^{2} c_{{{\text{Dio}}(x_{\text{Dio}} = 1)}}^{'} }},$$

15 $$S_{{{\text{L}} - {\text{Dio}}}} = \frac{{\left( {S_{\text{L}} S_{\text{Dio}} + c_{{{\text{L}}(x_{\text{Dio}} = 0)}}^{'} c_{{{\text{Dio}}(x_{\text{Dio}} = 1)}}^{'} S_{\text{L}}^{ 2} S_{\text{Dio}}^{2} } \right)\left( {S_{\text{L}} + S_{\text{Dio}} } \right)}}{{S_{\text{L}}^{ 3} c_{{{\text{L}}(x_{\text{Dio}} = 0)}}^{'} + S_{\text{Dio}}^{ 3} c_{{{\text{Dio}}(x_{\text{Dio}} = 1)}}^{'} }}.$$

The slopes of tangent lines at the points (pure component L) *x*_Dio_ = 0 and *x*_Dio_ = 1 (pure component Dio) may be calculated from the following equations (Petelska and Figaszewski 2009; Serafin et al. 2015):

16 $$c_{{{\text{L}}(x_{\text{Dio}} = 0)}}^{'} = \frac{{K_{{{\text{L}} - {\text{Dio}}}} \left( {S_{\text{L}} - S_{{{\text{L}} - {\text{Dio}}}} } \right) - S_{\text{L}} S_{\text{Dio}} }}{{S_{\text{L}}^{2} \left( {S_{\text{L}} + K_{{{\text{L}} - {\text{Dio}}}} } \right)}},$$

17 $$c_{{{\text{Dio}}(x_{\text{Dio}} = 1)}}^{'} = \frac{{ - K_{{{\text{L}} - {\text{Dio}}}} \left( {S_{\text{Dio}} - S_{{{\text{L}} - {\text{Dio}}}} } \right) - S_{\text{L}} S_{\text{Dio}} }}{{S_{\text{Dio}}^{ 2} \left( {K_{{{\text{L}} - {\text{Dio}}}} - S_{\text{Dio}} } \right)}}.$$

Equations (16) and (17) may be used for verification of slopes obtained either from theory or by experiment. Agreement between the slopes indicates that the method of calculating *K*_L–Dio_ and *S*_L–Dio_ is justified.

Using the values calculated for *S*_L–Dio_ and *K*_L–Dio_ in Eqs. (14) and (15), theoretical ${\text{c}}_{\text{L}}^{\prime }$ and ${\text{c}}_{\text{Dio}}^{\prime }$ values were calculated and compared with the slopes of lines tangent to the experimental data at points *x*_Dio_ = 0 and *x*_Dio_ = 1.

Theoretical values were calculated from the system of Eqs. (1)–(3). Attempts to solve this system of equations resulted in the final equation presented above.

18 $$a_{\text{L}} = \frac{{1 - c_{\text{L}} S_{\text{L}} - c_{\text{Dio}} S_{\text{Dio}} }}{{S_{{{\text{L}} - {\text{Dio}}}} - S_{\text{Dio}} - S_{\text{L}} }},$$

19 $$a_{\text{Dio}} = \frac{{c_{\text{L}} S_{{{\text{L}} - {\text{Dio}}}} - c_{\text{L}} S_{\text{Dio}} + c_{\text{Dio}} S_{\text{Dio}} - 1}}{{S_{{{\text{L}} - {\text{Dio}}}} - S_{\text{Dio}} - S_{\text{L}} }},$$

20 $$a_{{{\text{L}} - {\text{Dio}}}} = \frac{{c_{\text{Dio}} S_{{{\text{L}} - {\text{Dio}}}} - c_{\text{Dio}} S_{\text{L}} - 1 + c_{\text{L}} S_{\text{L}} }}{{S_{{{\text{L}} - {\text{Dio}}}} - S_{\text{Dio}} - S_{\text{L}} }}.$$

Next, the obtained *a*_L_, *a*_Dio_, *a*_L–Dio_ values are substituted into the Eqs. (2) and (3) to calculate the parameters *c*_L_ and *c*_Dio_ which were presented as a line in figures (Figs. 4, 5 in Results and Discussion section).

The lipid (PC or Ch)—Dio complex formation energy was calculated from Eq. (21):

21 $$- \log K = \frac{{\Delta G^{0} }}{2.3RT},$$

where *K* (m^2^ mol^−1^) is the stability constant of lipid–Dio complex; Δ*G*^0^ (J mol^−1^) is the lipid–Dio complex formation energy; *R* (J mol^−1^ K^−1^) is the gas constant; *T* (K) is the temperature.

## Materials and Methods

### Materials

PC from soybean (≥97 %, Roth) prepared by a modification of the procedure of Singleton et al. (1965) was used in the experiment. Dio ≥99 % (TLC) and Ch from hog liver ≥97 % (GC) were purchased from Fluka and were used as received. The molecular weights of the PC, Dio, and Ch were approximately 752.08, 386.67, and 386.67 g mol^−1^, respectively. The 1-chloropropane solvent (>98 % pure) was supplied by Aldrich.

Solutions were prepared by dissolving appropriate amounts of each material in 1-chloropropane at a concentration of 1 mg cm^−3^ and were stored at 4 °C.

The water used in the experiments was prepared by triple distillation (the second distillation was performed over KMnO_4_ and KOH to remove organic impurities).

### Methods

The homemade computer-controlled apparatus used for surface tension measurements was presented in previous paper (Petelska and Figaszewski 2009).

The surface tension measurements were carried out at the water/air interface at 22 °C and were expressed as surface pressure–area per molecule (π–A) isotherms. For all experiments, the trough was filled with triple-distilled water as the subphase. The monolayers were prepared by spreading a defined volume of a lipid solution in 1-chloropropane on the aqueous subphase using a Hamilton micro-syringe. Ten minutes were allowed for solvent evaporation and monolayer equilibration before an experiment was begun. The monolayer was continuously compressed to obtain the π–A isotherms using the glass barrier. The Nima ST9002 computer program was used to calculate the surface pressure of the monolayer π as a function of surface area per molecule A: π = γ_0 −_γ = *f*(A), where γ_0_ is the surface tension of the bare air/water interface and γ is the surface tension of the lipid-covered surface.

Before each trial, the Teflon trough (trough size 648 cm^2^) was washed and rinsed with purified water. The subphase surface was cleaned just prior to each measurement by suction with a vacuum pump until the surface tension was constant and equal to the surface tension value of pure water at 22 °C (approximately 72 m N m^−1^). All glasswares in contact with the samples were cleaned with chromic acid and repeatedly rinsed with purified water before use.

The system was enclosed in an acrylic box to minimize water evaporation, to ensure high humidity, and to avoid contamination of the system.

All of the reported values are highly reproducible and represent the average of at least five experiments. The standard deviation for surface area measurements was <1 %.

## Results and Discussion

In this paper, we present evidence for the formation of 1:1 PC–Dio and Ch–Dio complexes at the air/water interface. The stability constants and surface occupied by the PC–Dio and Ch–Dio complexes were calculated using equations from Theory section.

**Fig. 2:**
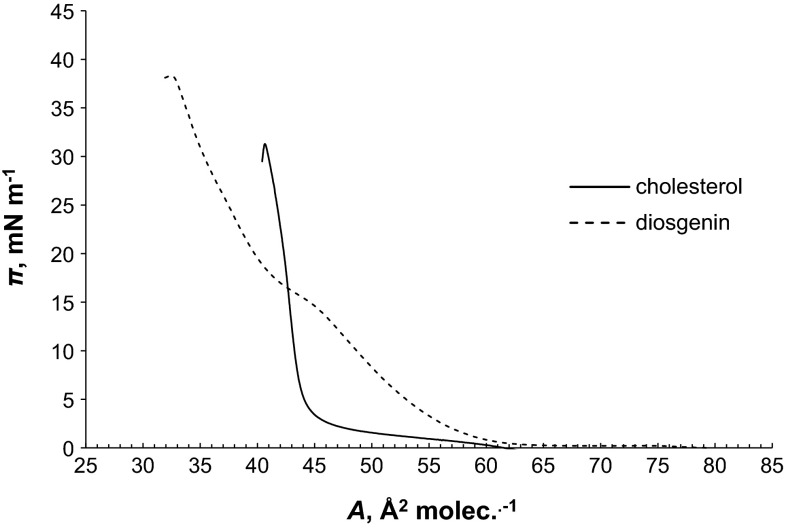
Isotherm of PC

Figure 2 presents π–A isotherms of Ch and Dio. Isotherms start at a very low surface pressure, near 0 m N m^−1^. These low surface pressures are typical when the gas phase (G) is present. At low surface pressure, we observed coexistence between the G phase and liquid-condensed phase (LC), as shown in Fig. 2. The coexistence region for Ch monolayer ends at about 45–46 Å^2^ mol^−1^, where the isotherm shows a sharp pressure increase, to enter into the pure LC phase region. The slope of Ch isotherm is very high, indicating that compressibility of the surface film is small. The low compressibility and the sharp slope of the isotherm suggested that the Ch monolayer is rather rigid and there is little reorientation of the Ch molecules during compression.

Despite its bulky hydrocarbon structure and its plate-like steroid ring, Ch monolayer at the air/water interface is remarkably stable under dynamic conditions of compression. According to Ries and Swift (1989), Brzozowska and Figaszewski (2002), Sabatini et al. (2008), Petelska and Figaszewski (2009, 2011), the extrapolated molecular area of Ch is around 39–44 Å^2^ mol^−1^, which is approximately equal to that of two vertically oriented saturated fatty acid straight-chain molecules. The extrapolated molecular area of Ch molecule calculated from our result is 44 ± 0.5 Å^2^ mol^−1^, which is in good agreement with the earlier published data.

**Fig. 3:**
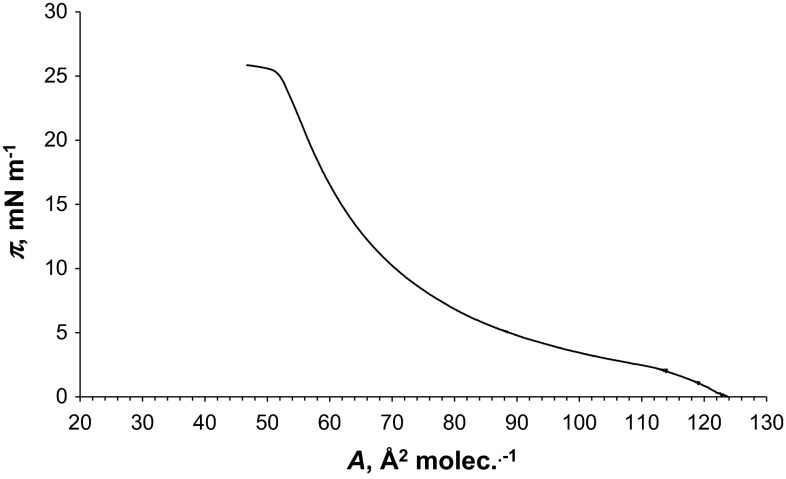
Isotherms of Dio and Ch

The π–A isotherms of Dio (Fig. 2) and PC (Fig. 3) are shaped differently. The PC formed stable monolayers, and its compression isotherm revealed a clear LE (liquid expanded) phase to LC phase main phase transition as reported previously (Brzozowska and Figaszewski 2002; Gzyl and Paluch 2001, 2004). Molecules in the gas phase are loosely packed at the water surface and behave like a 2D gas. As the monolayer is being compressed, there is a G–LE transition. In the LE phase, molecules behave like a 2D liquid and are not as free to move about as it is observed in the gas phase. As the monolayer is being compressed further, the LE–LC phase transition will occur. The LE–LC phase transition occurs at ∼27 m N m^−1^ for PC and about 10 m N m^−1^ higher than that of Dio.

The surface areas for the Dio and PC molecules are 45 ± 0.5 Å^2^ mol^−1^ equal and 75 ± 0.8 Å^2^ mol^−1^, respectively. These values were obtained experimentally by extrapolating the isotherms to π = 0. This is a standard procedure for the determination of this value presented in the paper concerning the monolayer experiments (Birdi 1989). The surface areas per lipid molecule we determined after the Langmuir trough is completely covered with the lipid molecules. Further reduction of the surface not have to immediately lead to the collapse or destruction of the monolayer. This is the minimum area to which the molecules can be compressed on the water surface without collapsing the monolayer. The orientation state of the molecules in a phase can be estimated quantitatively by comparing the extrapolated area per molecule with that of molecule cross-sectional area in the bulk single crystal (Gupta and Manjuladevi 2012).

The value for PC is agreed with the reported values in literature 45–96 Å^2^ (Joos and Demel 1969; Jain 1972; Smondyrev and Berkowitz 1998; Tien and Ottova-Leitmannova 2003; Gzyl and Paluch 2001, 2004; Sovago et al. 2007; Petelska and Figaszewski 2009; Serafin et al. 2015).

### Ch–Dio Complex

**Fig. 4:**
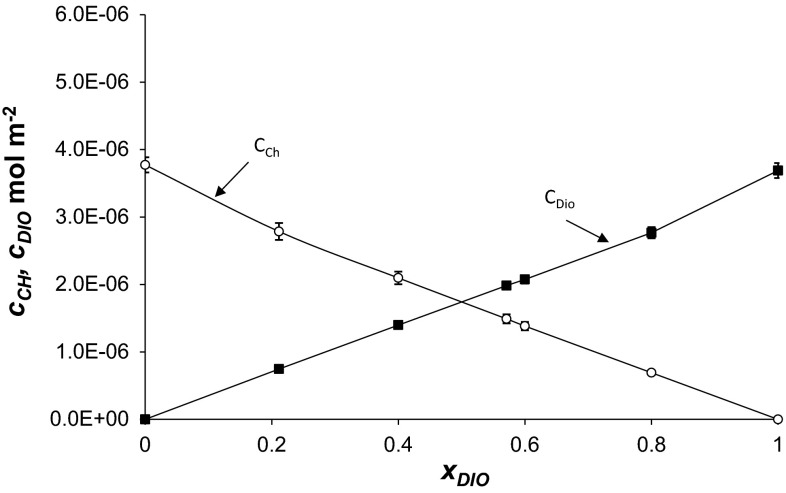
The dependence of total surface concentration of Ch (*c*_Ch_) and Dio (*c*_Dio_) on the mole fraction of Dio (the experimental values are indicated by *points* and the theoretical values by the *curve*)

The total surface concentrations of Ch (*c*_Ch_) and Dio (*c*_Dio_) versus mole fraction of Dio are presented in Fig. 4. The nearly linear shape of the *c*_Ch_ = *f (x*_Ch_*)* function confirms the condensed character of the membrane (Birdi 1989). The observed decrease in surface area per phospholipid molecule indicates a condensation effect induced by cholesterol (Yeagle 1985). It is remarkable that the function *c*_Ch_ = *f(x*_Ch_*)* is almost linear for *x*_Ch_ > 0.5.

Such interactions in the investigated Ch–Dio system can be explained in terms of complexes (Schöpke and Hiller 1990; Sędek and Michalik 2005). The 1:1 Ch–Dio complex has been assumed to exist in monolayers composed of Ch and Dio (Eqs. 1–5). It is characterized by the stability constant, *K*_*AB*_ (Eq. 4).

The stability constant, *K*_Ch–Dio_ = 1.37 × 10^6^ m^2^ mol^−1^, and the area per Ch–Dio complex, *S*_Ch–Dio_ = 5 × 10^5^ m^2^ mol^−1^ (83 ± 0.8 Å^2^ mol^−1^), were calculated by inserting the experimental data into Eqs. (6) and (7).

The *S*_Ch–Dio_ value obtained this way is higher than the area of a Dio molecule, *S*_Dio_ = (45 ± 0.5 Å^2^ mol^−1^), but slightly lower than the sum of the areas of Ch and Dio (*S*_Ch_ + *S*_Dio_ = 89 ± 0.9 Å^2^).

The complex formation energy (Gibbs free energy) values for the Ch–Dio complex calculated from Eq. (10) is equal −34.97 ± 1.75 kJ mol^−1^.

### PC–Dio Complex

**Fig. 5:**
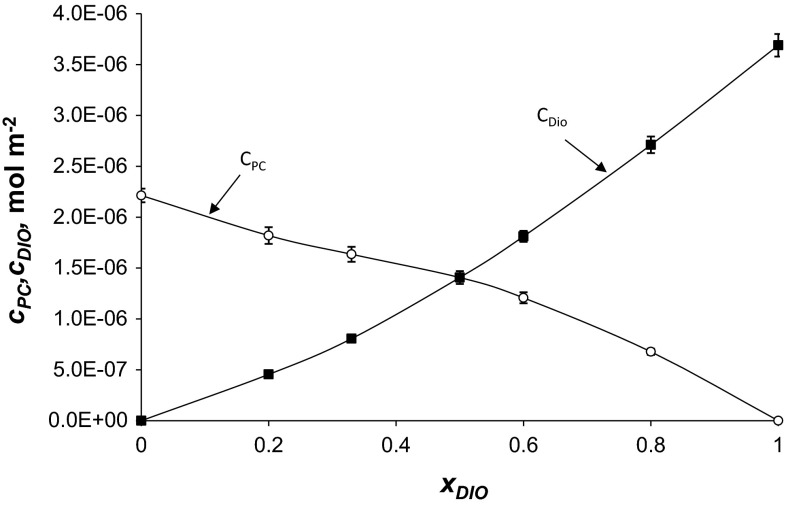
The dependence of total surface concentration of PC (*c*_PC_) and Dio (*c*_Dio_) on the mole fraction of Dio (the experimental values are indicated by *points* and the theoretical values by the *curve*)

Figure 5 presents the total surface concentration of PC (cPC) and Dio (cDio) versus mole fraction of Dio. In monolayers composed of PC and Dio (Eqs. 1–5) has been assumed to exist the 1:1 PC–Dio complex. This complex is characterized by the stability constants, *K*_PC–Dio_ (Eq. 4), which was equal *K*_PC–Dio_ = 6.46 × 10^5^ m^2^ mol^−1^. The stability constant presented above was calculated by inserting the experimental data into Eq. (6). The area per PC–Dio complex, *S*_PC–Dio_ = 7.89 × 10^5^ m^2^ mol^−1^ (131 ± 1.3 Å^2^ mol^−1^), was calculated by inserting the experimental data into Eq. (7). The *S*_PC–Dio_ value for PC–Dio complex obtained in this way is higher than the area of a PC molecule *S*_PC_ = (75 ± 0.8 Å^2^ mol^−1^) and slightly higher than the sum of the areas of PC and Dio (*S*_PC_ + *S*_Dio_ = 120 ± 1.3 Å^2^).

The complex formation energy (Gibbs free energy) values for the PC–Dio complex calculated from Eq. (10) is equal −32.78 ± 1.54 kJ mol^−1^.

In Figs. 4 and 5, the experimental points are compared with the values calculated using Eqs. 1–3 (depicted as lines). The theoretical values obtained are presented in Figs. 4 and 5 and are marked by lines; points on the same figure show the experimental value systems. It can be seen that the agreement between experimental and theoretical points is very good, which verifies the assumption of the formation of the 1:1 complexes in the mixed PC–Dio and Ch–Dio monolayers. The lack of variation between theoretical and experimental point indicates that theoretical model (presented in Theory section) is sufficient to describe the interaction in lipid–Dio systems.

Table 1 lists several physicochemical parameters for monolayers containing Ch–Dio and PC–Dio complexes.

**Table 1:** Selected physicochemical parameters for complexes: PC–Dio and Ch–Dio

Examined system	Surface area occupied by one molecule of complex (Ǻ^2^ mol^−1^)	Stability constant of examined complex m^2^ mol^−1^)	Complex formation energy (Gibbs free energy) (kJ mol^−1^)
Ch–Dio	83 ± 0.8	1.37 × 10^6^	−34.97 ± 1.75
PC–Dio	131 ± 1.3	6.46 × 10^5^	−32.78 ± 1.54

Nontoxic bioactive compounds (e.g., Dio) capable of inhibiting the neoplastic process have been the subject of intensive recent research. Increasing attention is being devoted to the design of functional foods with therapeutic or preventive action towards specific diseases or tumor types. Cellular and animal models, as well as clinical tests, have identified that certain natural compounds commonly consumed by humans have the potential ability to prevent and inhibit cancer development. Understanding the interactions of such compounds with pathologically changed cell membranes is essential for understanding their impact on the course of a complex series of biochemical and physicochemical processes. Our research focuses on phenomena occurring in both biological membranes as well as in model membranes (lipid monolayers). Such investigations will aid progress in medical and pharmaceutical science.
